# Localised bone grafting of acetabular cysts during total hip replacement

**DOI:** 10.1308/003588412X13373405387050h

**Published:** 2012-10

**Authors:** A Mumith, P Ward

**Affiliations:** Dorset County Hospital NHS Foundation Trust,UK

**Figure 1:**
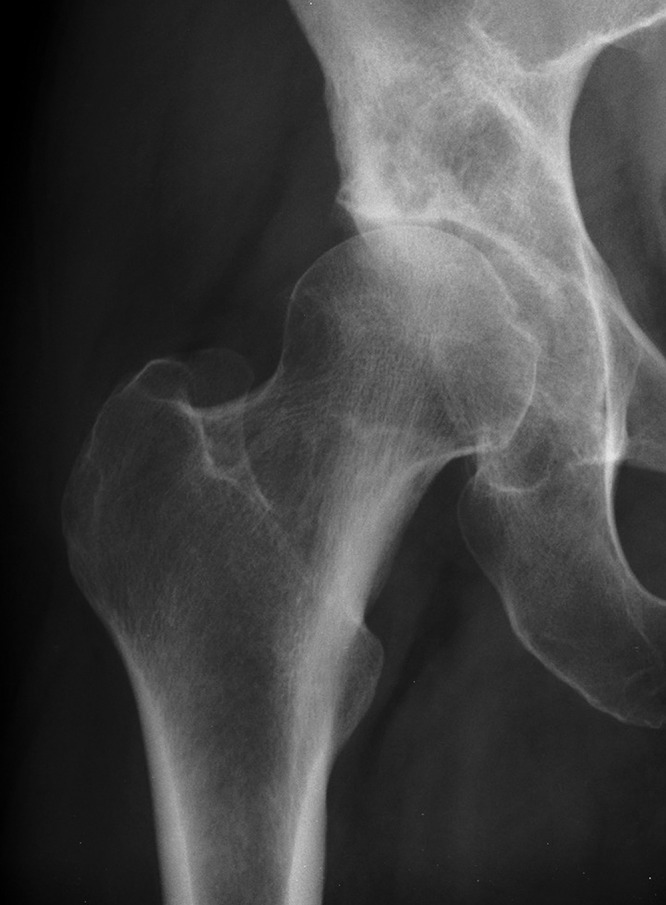
Anteroposterior x-ray of right hip with a superiorly located acetabular cyst

**Figure 2:**
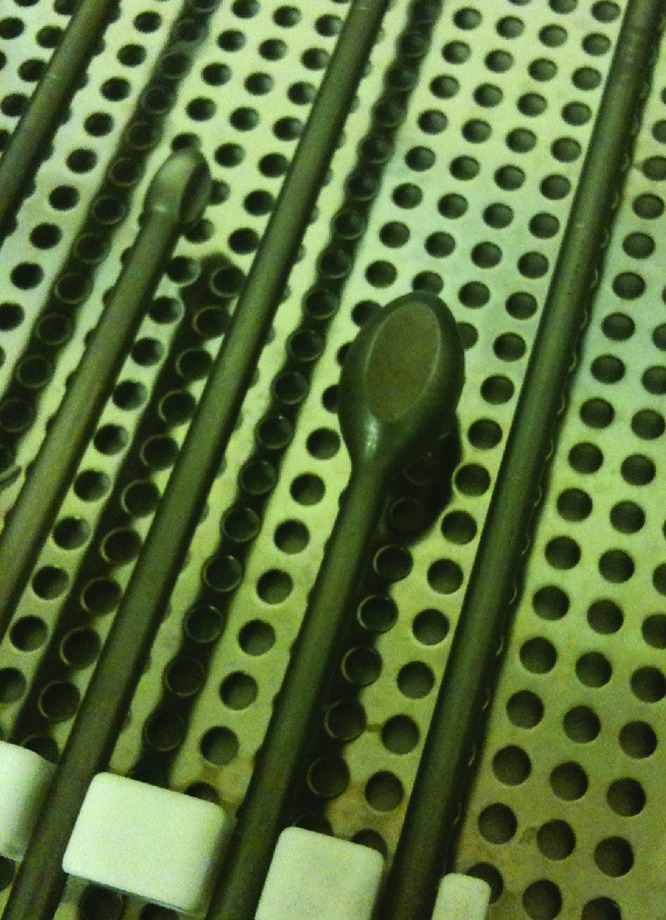
Exeter™ plug trial head is ideal for bone graft impaction within cyst

Bone cysts are commonly found when preparing the acetabulum during a total hip replacement (Fig 1). We describe a simple yet effective technique of localised bone grafting using the Exeter™ (Stryker, Newbury, UK) plug trials already available when using the Exeter™ total hip system (Fig 2).

The Exeter™ plug trials used to measure cement restrictors can be employed to pack bone graft into cysts. They are supplied in diameters from 6mm to 20mm and allow the surgeon to choose the appropriate sized plug trial to fit the bone cyst for optimal impaction. The long T-handle allows this technique to also be applied in overweight patients with deeply located acetabuli.

